# Recurrent subcutaneous human Dirofilariasis due to *Dirofilaria repens *after surgical removal of the worm and anthelmintic treatment

**DOI:** 10.1186/1756-3305-7-S1-P3

**Published:** 2014-04-01

**Authors:** M Lupșe, V Mircean, A Cavasi, AD Mihalca

**Affiliations:** 1 University of Medicine and Pharmacy "Iuliu Haţieganu" Cluj-Napoca, Romania; 2 University of Agricultural Sciences and Veterinary Medicine, Cluj-Napoca, Romania

## 

The genus *Dirofilaria *includes various species that are natural parasites of dogs, cats, foxes, and wild mammals, transmitted by mosquito vectors. *Dirofilaria repens *is commonly encountered in the subcutaneous tissue of dogs, foxes, and cats. Due to recent increase of human *Dirofilaria repens *it is considered an emerging zoonosis. In Romania, there are only few reports regarding the infection with *D. repens *in dogs and in humans. Patients usually present single migratory submucosal or subcutaneous nodule which may or may not be tender. Surgical excision of lesions and affected areas is the treatment of choice for dirofilariasis. Anthelmintics are not usually recommended.

We describe the case of a 65 years old, retired woman, without history of traveling in the past years, living in the Oradea (north-west part of Romania), in an urban area, who presented a left breast subcutaneous nodule with fever and eosinophilia in August 2012. After surgical excision, histopathology and PCR assay (Isolate Genomic DNA Kit, Bioline, UK using 12S ADNr gene (250 bp) the diagnosis of *D. repens *infection was established. Patient received Albendazole 400 mg/day for 7 days after surgery. The eosinophil count became normal and no symptoms were noticed till December 2012 when another nodular lesion developed in the left breast, accompanied by eosinophilia. After the surgical removal of the second nodule, another larval worm was identified as *D. repens*. The patient was treated with 150 μg/kg BW of ivermectin and no other nodules appeared. Eosinophiles level remained normal during one year follow-up. This is the first report of autochthonous *D. repens *infection in humans in the northern part of the country and, to our knowledge, the first case of recurrent subcutaneous human dirofilariasis. The recurrence in this case is most probably the result of infection with multiple larvae, of which at least two developed into nodules at four month interval, rather than a repeated infection. We recommend the treatment with ivermectin after surgical excision to avoid recurrences in dirofilariasis.

